# A new large-scale meta-epidemiological study on bias in randomized trials using routinely collected risk-of-bias assessments by cochrane reviewers: results from the robes study

**DOI:** 10.1186/1745-6215-16-S2-P168

**Published:** 2015-11-16

**Authors:** Jelena Savovic, Rebecca Turner, David Mawdsley, Julian Higgins, Jonathan Sterne

**Affiliations:** 1 University of Bristol, Bristol, UK; 2 MRC Biostatistics Unit, Cambridge, UK

## Introduction

Empirical evidence suggests that certain aspects of trial design may lead to biased intervention effect estimates. We examined the influence of risk-of-bias judgements from Cochrane reviews for sequence generation, allocation concealment, blinding and incomplete data on intervention effect estimates in a large collection of meta-analyses (MAs).

## Methods

We selected MAs with dichotomous outcomes and > 4 included trials from intervention reviews with fully completed risk-of-bias tool, published in issue 4, 2011 of the Cochrane Library. We classified outcome measures as mortality, other objective or subjective, and estimated the effect of risk-of-bias domain judgements on average bias (ratios of odds ratios [ROR] with 95% credible intervals [Cr-I]) using Bayesian hierarchical models.

## Results

Among 2815 trials in 256 meta-analyses, intervention effect estimates were on average exaggerated in trials with high or unclear risk-of-bias (versus low) for random sequence generation (ROR 0.91 [95% Cr-I 0.86, 0.98]), for allocation concealment (ROR 0.92 [95% Cr-I 0.86-0.98]) and for blinding (ROR 0.87 [95% Cr-I 0.80, 0.93]). Unlike our previous study, we did not observe consistently different bias or between-trial heterogeneity in bias in MAs with subjective outcomes compared to mortality. Results from analyses of the influences of incomplete data were inconclusive.

## Limitations

Possible inconsistency in criteria for risk-of-bias judgments applied by individual reviewers is a likely limitation of routinely collected bias assessments.

## Conclusions

Inadequate randomization or lack of blinding may lead to exaggeration of intervention effect estimates in trials, but it is unclear if this effect differs by outcome type.

